# Fish-T1K (Transcriptomes of 1,000 Fishes) Project: large-scale transcriptome data for fish evolution studies

**DOI:** 10.1186/s13742-016-0124-7

**Published:** 2016-05-03

**Authors:** Ying Sun, Yu Huang, Xiaofeng Li, Carole C. Baldwin, Zhuocheng Zhou, Zhixiang Yan, Keith A. Crandall, Yong Zhang, Xiaomeng Zhao, Min Wang, Alex Wong, Chao Fang, Xinhui Zhang, Hai Huang, Jose V. Lopez, Kirk Kilfoyle, Yong Zhang, Guillermo Ortí, Byrappa Venkatesh, Qiong Shi

**Affiliations:** State Key Laboratory of Biocontrol, Institute of Aquatic Economic Animals and Guangdong Provincial Key Laboratory for Aquatic Economic Animals, School of Life Sciences, Sun Yat-Sen University, Guangzhou, 510275 China; Shenzhen Key Lab of Marine Genomics, Guangdong Provincial Key Lab of Molecular Breeding in Marine Economic Animals, BGI, Shenzhen, 518083 China; National Museum of Natural History, Smithsonian Institution, Washington, DC 20560 USA; China Fisheries Association, Beijing, 100000 China; China National Genebank, Shenzhen, 518083 China; Department of Biological Sciences, The George Washington University, Washington, DC 20052 USA; BGI-Zhenjiang Institute of Hydrobiology, Zhenjiang, 212000 China; BGI-Hong Kong, Hong Kong, 999077 China; Sanya Science and Technology Academy for Crop Winter Multiplication, Hainan, 572000 China; Oceanographic Center, Nova Southeastern University, Fort Lauderdale, 33004 USA; Institute of Molecular and Cell Biology, A*STAR, Singapore, 138673 Singapore; College of Life Sciences, Shenzhen University, Shenzhen, 518060 China

**Keywords:** Fish-T1K, Fish, Transcriptome, RNA, Database, Biodiversity

## Abstract

Ray-finned fishes (Actinopterygii) represent more than 50 % of extant vertebrates and are of great evolutionary, ecologic and economic significance, but they are relatively underrepresented in ‘omics studies. Increased availability of transcriptome data for these species will allow researchers to better understand changes in gene expression, and to carry out functional analyses. An international project known as the “Transcriptomes of 1,000 Fishes” (Fish-T1K) project has been established to generate RNA-seq transcriptome sequences for 1,000 diverse species of ray-finned fishes. The first phase of this project has produced transcriptomes from more than 180 ray-finned fishes, representing 142 species and covering 51 orders and 109 families. Here we provide an overview of the goals of this project and the work done so far.

**Electronic supplementary material:**

The online version of this article (doi:10.1186/s13742-016-0124-7) contains supplementary material, which is available to authorized users.

## Background

Ray-finned fishes (Actinopterygii) are the most diverse and abundant group of extant vertebrates. Thus far, approximately 32,900 fish species are recorded in FishBase [1]. Fishes encompass enormous variation in morphology, physiology and ecology. They are of great economic and medical significance as a primary source of protein for people worldwide, as a novel source of active ingredients in pharmaceuticals [2], and as evolutionary models for specific human diseases and conditions [3].

However, genomic resources for fishes are relatively underrepresented and published genetic data represent only a small fraction of extant fish species. So far, the whole genomes of only 38 fish species have been published (Additional file 1) and, although the number is growing (Additional file 2), searching the National Center for Biotechnology Information (NCBI)’s Sequence Read Archive (SRA) database for “fish AND transcriptome” yields 16,975 transcriptomes of only 242 fish species (Table 1). A lack of genomic resources for most fish species motivated us to generate large-scale fish transcriptome data and establish a database that may be used by scientists around the world. To this end, we initiated the “Transcriptomes of 1,000 Fishes” (Fish-T1K) project, an effort devoted to sequencing the transcriptomes of 1,000 different species of ray-finned fishes.

**Table 1:** List of fish species with published transcriptome data in NCBI’s SRA, and those generated by Fish-T1K

Order	No. of species in SRA	No. of species in Fish-T1K	No. of new species generated by Fish-T1K
Cypriniformes	42	5	3
Cyprinodontiformes	33	2	0
Perciformes	21	9	9
Cichliformes	15	2	2
Salmoniformes	14	0	0
Order-level *incertae sedis* in Eupercaria	9	2	2
Pleuronectiformes	9	2	1
Osteoglossiformes	8	4	2
Siluriformes	8	9	8
Clupeiformes	6	1	1
Syngnathiformes	6	5	5
Gymnotiformes	5	2	1
Acipenseriformes	4	1	1
Anabantiformes	4	4	4
Anguilliformes	4	4	3
Centrarchiformes	4	4	3
Scombriformes	4	2	2
Beloniformes	3	2	2
Characiformes	3	6	6
Gadiformes	3	1	1
Order-level *incertae sedis* in Ovalentaria	3	5	5
Tetraodontiformes	3	4	4
Carangiformes	2	2	1
Amiiformes	1	1	0
Batrachoidiformes	1	1	1
Blenniiformes	1	4	4
Esociformes	1	0	0
Labriformes	1	3	2
Lepisosteiformes	1	2	2
Ophidiiformes	1	2	2
Osmeriformes	1	0	0
Pempheriformes	1	2	2
Polypteriformes	1	3	3
Spariformes	1	2	2
Synbranchiformes	1	2	2
Argentiniformes	0	1	1
Atheriniformes	0	2	2
Aulopiformes	0	2	2
Chaetodontiformes	0	1	1
Elopiformes	0	1	1
Ephippiformes	0	2	2
Galaxiiformes	0	1	1
Gobiiformes	0	3	3
Holocentriformes	0	3	3
Kurtiformes	0	2	2
Lepidogalaxiiformes	0	1	1
Lobotiformes	0	1	1
Lophiiformes	0	2	2
Mugiliformes	0	2	2
Order-level *incertae sedis* in Carangimorphariae	0	3	3
Order-level *incertae sedis* in Percomorpharia	0	12	12
Percopsiformes	0	1	1
Uranoscopiformes	0	1	1
Zeiformes	0	1	1
others (Chondrichthyes and Sarcopterygii)	17	/	/
All	242	142	128

### Fish-T1K

Fish-T1K is an international, collaborative and non-profit initiative officially launched by BGI and the China National Genebank (CNGB) in November 2013. The objective is to generate RNA-seq transcriptome sequences for 1,000 diverse fish species to help scientists unravel the mysteries of fish evolution, and pursue innovative approaches and strategies for addressing challenges in fish breeding, disease control and prevention, seafood safety, and biodiversity conservation.

Through this project, an integrated biobank will be established, incorporating a high-level bio-repository and a large-scale transcriptome database. The biobank will collect and store fish genetic resources including vouchers and frozen tissues, DNA and RNA nucleotides, together with related sample information documented according to standard operating procedures (SOPs). A companion database, committed to being the world’s largest database of fish transcriptomes, has already been established and provides access to the sequences via BLAST search.

### The Fish-T1K consortium

**Fig. 1:**
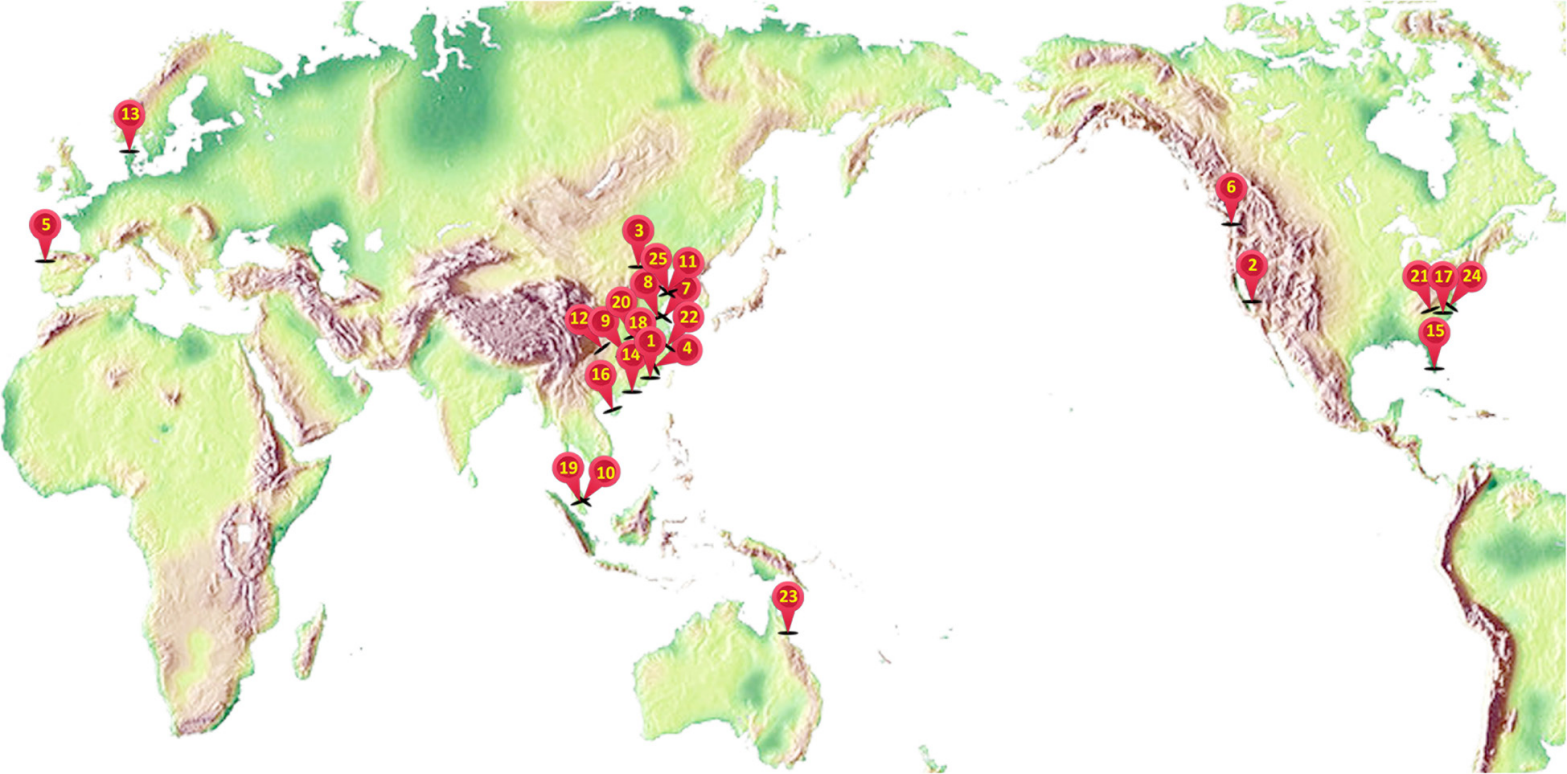
Distribution of Fish-T1K Consortium members. See detailed information of these numbered institutions in Additional file 3

More than 40 scientists from 25 institutions across seven countries are active members of the Fish-T1K project (Fig. 1; Additional file 3). The Steering Committee consists of six core consortium members who are recognized experts in ichthyology, taxonomy, bioinformatics, phylogenetics, and evolution. In addition to the head office at BGI in Shenzhen, China, we have also established a hub at the Smithsonian National Museum of Natural History (NMNH) in Washington DC, USA, to facilitate quality sample collection from North America.

### Species selection

Fish-T1K proposes to sequence 1,000 different ray-finned fish species representing all the orders and major families [4], and filling important gaps in the phylogenetic tree. Species that are endangered, of great economic and medical significance, or exhibit extreme phenotypes will also be targeted. Candidate species will be decided based on their importance and availability, while the target number will be a compromise between scientific needs and practical limitations such as financial constraints and availability of specimens.

### Subprojects

To maximize usage of these transcripts, Fish-T1K has launched several subprojects to address specific questions in fish evolution. The major research goal of Fish-T1K is to reconstruct a comprehensive molecular phylogeny of ray-finned fishes to further resolve and test existing phylogenetic hypotheses. Additional subprojects include analysis of the evolutionary genomics of fish venoms, evolution of the annual life cycle in killifishes, and adaptations related to marine-to-freshwater transitions/migration.

### SOPs and best practices

In the past two years, the Fish-T1K Team has established a series of SOPs, approved by BGI’s Institutional Review Board on Bioethics and Biosafety (No. BGI-IRB 15139), to ensure high quality sampling is achieved. Adhering to these SOPs means that all of our genetic resources, data and associated metadata are appropriately obtained, documented, and stored, which is helpful in establishing and optimizing standards common to large-scale transcriptome and genome sequencing projects.

Transcriptome data from multiple tissues of five fishes were generated as a pilot quality control test (Additional file 4). Accordingly, total RNA is now routinely extracted from gills and other tissues of interest, and approximately 3.5 Gb of raw data are generated for each sample. Clean reads are assembled *de novo* into contigs with SOAPdenovo-Trans (v1.3) [5], and the final assembled transcripts are used for annotation, ortholog prediction and other analyses.

### Current RNA sequencing progress

The Fish-T1K team has established a collaborative global network for collecting specimens. As of January 2016, 7,000 high quality fish samples were collected from Australia, the Caribbean, Denmark, Singapore, the UK, USA, and many places in China such as the Tibetan Plateau, Sanya, and the Yellow Sea. From these 7,000 samples, RNA samples were extracted from 142 ray-finned species covering 51 orders and 109 families, and around 180 transcriptomes have been produced (Table 1; Additional file 5). Meanwhile, more RNA samples from other species are being isolated and sequenced.

### Website and database

The official Fish-T1k website [6] is equipped with a database for BLAST search. The website provides detailed information about the Fish-T1K project, and particular sample information (RNA quality, sample provider, etc.) and data quality (raw data size, scaffold size and number, etc.) are presented in the database. Users can access the BLAST tool and download sequences of interest. Data will be uploaded periodically as sample collection and transcriptome sequencing progresses.

### Data sharing policy and data availability

All sequences generated from Fish-T1K will be deposited in NCBI and GigaDB in addition to the Fish-T1K database, following the Fort Lauderdale rules [7] and Toronto International Data Release Workshop guidelines [8], and will be released at least in the time of publication of any resulting papers. We plan to peer review and publish the SOP and method papers, will be published and we’re expecting publications for some of the ongoing subprojects are also expected in one the coming year or sooner.

### Fish-T1K membership

All are welcome to participate in Fish-T1K and to propose new subprojects; these should address a major question in fish evolution and lead to (a) significant publication(s). Interested researchers can email fisht1k@genomics.cn with a brief proposal. The significance, question(s) to be addressed and fishes/tissues to be sequenced and analyzed should be included. On acceptance of a proposal, the lead scientist(s) will be asked to collect any fish tissues that are not already in our list, and to be in charge of analyzing and publishing the generated data.

## Conclusions

Similar initiatives already exist to sequence the transcriptomes of large numbers of plants (1KP [9]) and insects (1KITE [10]). They have been well received and have been useful in establishing Fish-T1K. Although some progress has already been made, the Fish-T1K is at an early stage. We will continue to expand the scope of the project: in the first phase we aim to cover all orders, and all families in the second phase. More species will be added as required by subprojects. As the world’s first large-scale transcriptome database exclusively for fish, Fish-T1K will greatly enhance the study of fish biology, and eventually contribute efforts towards global fish biodiversity conservation and the sustainable utilization of natural fish resources.

## Additional files

Additional file 1: List of fishes with published genome data. (DOCX 30 kb) Additional file 2: Number of fish species with newly published transcriptomes in SRA of the NCBI from 2009 to 2015. (TIF 4045 kb) Additional file 3: List of the current Fish-T1K Consortium members. (DOCX 19 kb) Additional file 4: Transcriptome data of five species for quality control. (DOCX 20 kb) Additional file 5: List of fishes with transcriptome data generated by Fish-T1K. (XLSX 87 kb)

## Abbreviations

1KITE: 1 K Insect Transcriptome Evolution
1KP: 1000 Plants
Fish-T1K: transcriptomes of 1,000 Fishes
NGS: next-generation sequencing
RNA-seq: RNA sequencing
SOPs: standard operating procedures. SRA: Sequence Read Archive.
SRA: Sequence Read Archive
