# Supplementary material for: Fish-T1K (Transcriptomes of 1,000 Fishes) Project: large-scale transcriptome data for fish evolution studies
Source: Gigascience. 2016 May 3;5:18. doi: 10.1186/s13742-016-0124-7 (PMC4853854; doi:10.1186/s13742-016-0124-7)
Supplement: Additional file 1: — List of fishes with published genome data. (DOCX 30 kb) [file 13742_2016_124_MOESM1_ESM.docx]

**Additional file 1.** List of fishes with published genome data (updated Jan. 2016).

| **No.** | **Scientific name** | **Common name** | **Citation** |
| --- | --- | --- | --- |
| 1 | *Anguilla japonica* | Japanese eel | Henkel CV, et al. 2012^1^ |
| 2 | *Astatotilapia burtoni* | Burton’s mouthbrooder | Brawand D, et al. 2014^2^ |
| 3 | *Astyanax mexicanus* | Mexican tetra | McGaugh SE, et al. 2014^3^ |
| 4 | *Boleophthalmus* pectinirostris | Great blue-spotted mudskipper | You X, et al. 2014^4^ |
| 5 | *Ctenopharyngodon idellus* | Grass carp | Wang Y, et al. 2015^5^ |
| 6 | *Cynoglossus semilaevis* | Tongue sole | Chen S, et al. 2014^6^ |
| 7 | *Cyprinus carpio* | Common carp | Xu P, et al. 2014^7^ |
| 8 | *Danio rerio* | Zebrafish | Howe K, et al. 2013^8^ |
| 9 | *Dicentrarchus labrax* | European seabass | Tine M, et al. 2014^9^ |
| 10 | *Electrophorus electricus* | Electric eel | Gallant JR, et al. 2014^10^ |
| 11 | *Esox lucius* | Northern pike | Rondeau EB, et al. 2014^11^ |
| 12 | *Gadus morhua* | Atlantic cod | Star B, et al. 2011^12^ |
| 13 | *Gasterosteus aculeatus* | Three-spined stickleback | Jones FC, et al. 2012^13^ |
| 14 | *Ictalurus punctatus* | Channel catfish | Jiang Y, et al. 2013^14^ |
| 15 | *Larimichthys crocea* | Large yellow croaker | Wu C, et al. 2014^15^ |
| 16 | *Lates calcarifer* | Barramundi perch | Domingos JA, et al. 2015^16^ |
| 17 | *Maylandia zebra* | Zebra mbuna | Brawand D, et al. 2014^2^ |
| 18 | *Neolamprologus brichardi* | Lyretail cichlid | Brawand D, et al. 2014^2^ |
| 19 | *Nothobranchius furzeri* | Turquoise killifish | Harel I, et al. 2015^17^ |
| 20 | *Notothenia coriiceps* | Black rockcod | Shin SC, et al. 2014^18^ |
| 21 | *Oncorhynchus mykiss* | Rainbow trout | Berthelot C, et al. 2014^19^ |
| 22 | *Oreochromis niloticus* | Nile tilapia | Guyon R, et al. 2012^20^ |
| 23 | *Oryzias latipes* | Japanese medaka | Kasahara M, et al. 2007^21^ |
| 24 | *Periophthalmodon schlosseri* | Giant mudskipper | You X, et al. 2014^4^ |
| 25 | *Periophthalmus magnuspinnatus* | Giant-fin mudskipper | You X, et al. 2014^4^ |
| 26 | *Poecilia reticulata* | Guppy | Fraser BA, et al. 2015^22^ |
| 27 | *Pseudopleuronectes yokohamae* | Marbled flounder | Arthofer W, et al. 2015^23^ |
| 28 | *Pundamilia nyererei* | Flame back cichlid | Brawand D, et al. 2014^2^ |
| 29 | *Salmo salar* | Atlantic salmon | Davidson WS, et al. 2010^24^ |
| 30 | *Scartelaos* *histophorus* | Waking goby | You X, et al. 2014^4^ |
| 31 | *Scleropages formosus* | Asian bonytongue | Austin CM, et al. 2015^25^ |
| 32 | *Sinocyclocheilus anshuiensis* | Golden-line barbell (Anshui) | Yang J, et al. 2016^26^ |
| 33 | *Sinocyclocheilus grahami* | Golden-line barbell (Dianchi) | Yang J, et al. 2016^26^ |
| 34 | *Sinocyclocheilus rhinocerous* | Golden-line barbell (Xijiao) | Yang J, et al. 2016^26^ |
| 35 | *Takifugu rubripes* | Torafugu | Kai W, et al. 2011^27^ |
| 36 | *Tetraodon nigroviridis* | Spotted green pufferfish | Jiang Y, et al. 2013^28^ |
| 37 | *Thunnus orientalis* | Pacific bluefin tuna | Nakamura Y, et al. 2013^29^ |
| 38 | *Xiphophorus maculatus* | Southern platyfish | Schartl M, et al. 2013^30^ |

**References:**

1. Henkel CV, Dirks RP, de Wijze DL, et al. First draft genome sequence of the Japanese eel, Anguilla japonica. Gene. 2012; 511(2): 195-201.

2. Brawand D, Wagner CE, Li YI, et al. The genomic substrate for adaptive radiation in African cichlid fish. Nature. 2014; 513(7518): 375-381.

3. McGaugh SE, Gross JB, Aken B, et al. The cavefish genome reveals candidate genes for eye loss. Nat Commun. 2014; 5(2041-1723): 5307.

4. You X, Bian C, Zan Q, et al. Mudskipper genomes provide insights into the terrestrial adaptation of amphibious fishes. Nat Commun. 2014; 5: 5594.

5. Wang Y, Lu Y, Zhang Y, et al. The draft genome of the grass carp (Ctenopharyngodon idellus) provides insights into its evolution and vegetarian adaptation. Nat Genet. 2015; 47(6): 625-631.

6. Chen S, Zhang G, Shao C, et al. Whole-genome sequence of a flatfish provides insights into ZW sex chromosome evolution and adaptation to a benthic lifestyle. Nat Genet. 2014; 46(3): 253-260.

7. Xu P, Zhang XF, Wang XM, et al. Genome sequence and genetic diversity of the common carp, Cyprinus carpio. Nat Genet. 2014; 46(11): 1212-1219.

8. Howe K, Clark MD, Torroja CF, et al. The zebrafish reference genome sequence and its relationship to the human genome. Nature. 2013; 496(7446): 498-503.

9. Tine M, Kuhl H, Gagnaire P, et al. European sea bass genome and its variation provide insights into adaptation to euryhalinity and speciation. Nat Commun. 2014; 5: 5770.

10. Gallant JR, Traeger LL, Volkening JD, et al. Nonhuman genetics. Genomic basis for the convergent evolution of electric organs. Science. 2014; 344(6191): 1522-1525.

11. Rondeau EB, Minkley DR, Leong JS, et al., The genome and linkage map of the northern pike (Esox lucius): conserved synteny revealed between the salmonid sister group and the Neoteleostei. PLoS One. 2014; 9(7): e102089.

12. Star B, Nederbragt AJ, Jentoft S, et al. The genome sequence of Atlantic cod reveals a unique immune system. Nature. 2011; 477(7363): 207-210.

13. Jones FC, Grabherr MG, Chan YF, et al. The genomic basis of adaptive evolution in threespine sticklebacks. Nature. 2012; 484(7392): 55-61.

14. Jiang Y, Gao X, Liu S, et al. Whole genome comparative analysis of channel catfish (Ictalurus punctatus) with four model fish species. BMC Genomics. 2013; 14: 780.

15. Wu C, Zhang D, Kan M, et al. The draft genome of the large yellow croaker reveals well-developed innate immunity. Nat Commun. 2014; 5: 5227.

16. Domingos JA, Zenger KR, and Jerry DR. Whole-genome shotgun sequence assembly enables rapid gene characterization in the tropical fish barramundi, Lates calcarifer. Anim Genet. 2015; 46(4):468-469.

17. Harel I, Benayoun BA, Machado B, et al. A platform for rapid exploration of aging and diseases in a naturally short-lived vertebrate. Cell. 2015; 160(5): 1013-1026.

18. Shin SC, Ahn do H, Kim SJ, et al. The genome sequence of the Antarctic bullhead notothen reveals evolutionary adaptations to a cold environment. Genome Biol. 2014; 15(9): 468.

19. Berthelot C, Brunet F, Chalopin D, et al. The rainbow trout genome provides novel insights into evolution after whole-genome duplication in vertebrates. Nat Commun. 2014; 5: 3657.

20. Guyon R, Rakotomanga M, Azzouzi N, et al. A high-resolution map of the Nile tilapia genome: a resource for studying cichlids and other percomorphs. BMC Genomics. 2012; 13: 222.

21. mKasahara M, Naruse K, Sasaki S, et al. The medaka draft genome and insights into vertebrate genome evolution. Nature. 2007; 447(7145): 714-719.

22. Fraser BA, Künstner A, Reznick DN, et al. Population genomics of natural and experimental populations of guppies (Poecilia reticulata). Mol Ecol. 2015; 24(2): 389-408.

23. Arthofer W, Bertini L, Caruso C, et al. Genomic resources notes accepted 1 February 2015 - 31 March 2015. Mol Ecol Resour. 2015; 15(4): 1014-1015.

24. Davidson WS, Koop BF, Jones SJ, et al. Sequencing the genome of the Atlantic salmon (Salmo salar). Genome Biol. 2010; 11(9): 403.

25. Austin CM, Tan MH, Croft LJ, et al. Whole genome sequencing of the asian arowana (Scleropages formosus) provides insights into the evolution of ray-finned fishes. Genome Biol Evol. 2015; 7(10): 2885-2895.

26. Yang J, Chen X, Bai j, et al, The Sinocyclocheilus cavefish genome provides insights into cave adaptation. BMC Biol. 2016; 14(1): p1.

27. Kai W, Kikuchi K, Tohari S, et al. Integration of the genetic map and genome assembly of fugu facilitates insights into distinct features of genome evolution in teleosts and mammals. Genome Biol Evol. 2011; 3:424-442.

28. Jiang Y, Gao X, Liu S, et al. Whole genome comparative analysis of channel catfish (Ictalurus punctatus) with four model fish species. BMC Genomics. 2013; 14(780): 1471-2164.

29. Nakamura Y, Mori K, Saitoh K, et al. Evolutionary changes of multiple visual pigment genes in the complete genome of Pacific bluefin tuna. Proc Natl Acad Sci USA. 2013; 110(27): 11061-11066.

30. Schartl M, Walter RB, Shen Y, et al. The genome of the platyfish, Xiphophorus maculatus, provides insights into evolutionary adaptation and several complex traits. Nat Genet. 2013; 45(5): 567-572.
