# Supplementary figures and images for: Fish-T1K (Transcriptomes of 1,000 Fishes) Project: large-scale transcriptome data for fish evolution studies
Source: Gigascience. 2016 May 3;5:18. doi: 10.1186/s13742-016-0124-7 (PMC4853854; doi:10.1186/s13742-016-0124-7)

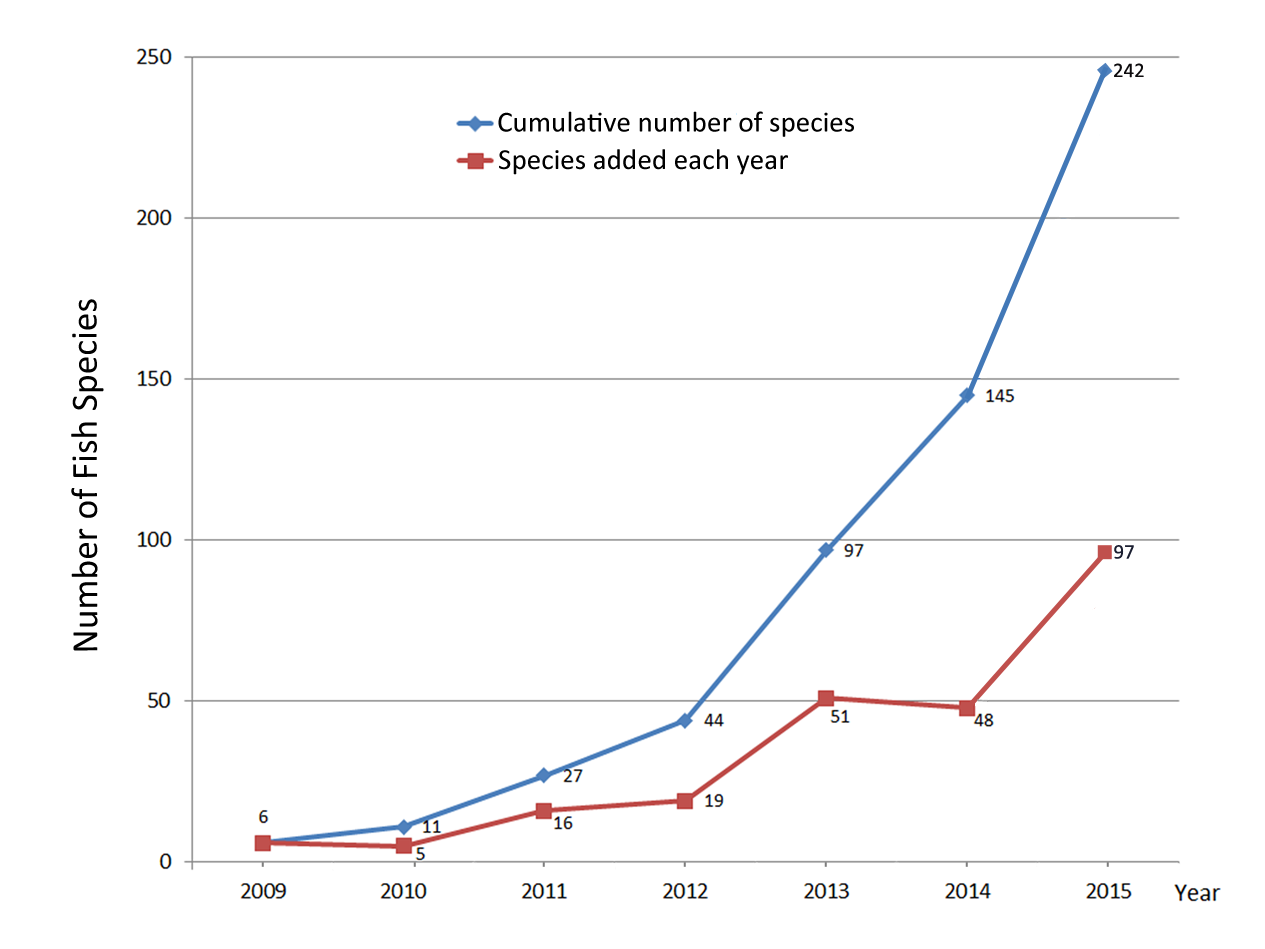

Supplement: Additional file 2: — Number of fish species with newly published transcriptomes in SRA of the NCBI from 2009 to 2015. (TIF 4045 kb) [file 13742_2016_124_MOESM2_ESM.tif]
