# Supplementary material for: Fish-T1K (Transcriptomes of 1,000 Fishes) Project: large-scale transcriptome data for fish evolution studies
Source: Gigascience. 2016 May 3;5:18. doi: 10.1186/s13742-016-0124-7 (PMC4853854; doi:10.1186/s13742-016-0124-7)
Supplement: Additional file 3: — List of the current Fish-T1K Consortium members. (DOCX 19 kb) [file 13742_2016_124_MOESM3_ESM.docx]

**Additional file 3.** List of the current Fish-T1K Consortium Members (updated in Jan. 2016).

| **No.** | **Department/Institute/University** | **City/State** | **Country** |
| --- | --- | --- | --- |
| 1 | BGI-Shenzhen | Shenzhen | China |
| 2 | Biodiversity Research Institute, Tulane University | Los Angeles | USA |
| 3 | China Fisheries Association | Beijing | China |
| 4 | China National Genebank | Shenzhen | China |
| 5 | CIIMAR, University of Porto | Porto | Portugal |
| 6 | Department of Biology, University of Victoria | Victoria | Canada |
| 7 | East China Sea Fisheries Research Institute, Chinese Academy of Fishery Sciences | Shanghai | China |
| 8 | Freshwater Fisheries Research Center, Chinese Academy of Fishery Sciences | Wuxi | China |
| 9 | Guangxi Mangrove Research Center | Beihai | China |
| 10 | Institute of Molecular and Cell Biology, A*STAR | Singapore | Singapore |
| 11 | Institute of Oceanology, Chinese Academy of Sciences | Qingdao | China |
| 12 | Kunming Institute of Zoology, Chinese Academy of Sciences | Yunnan | China |
| 13 | Natural History Museum of Denmark, University of Copenhagen | Copenhagen | Denmark |
| 14 | Ocean Park Hong Kong | Hong Kong | China |
| 15 | Oceanographic Center, Nova Southeastern University | Florida | USA |
| 16 | Sanya Science & Technology Academy for Crop Winter Multiplication | Hainan | China |
| 17 | Smithsonian National Museum of Natural History | Washington D.C | USA |
| 18 | South China Sea Institute of Oceanology, Chinese Academy of Sciences | Guangzhou | China |
| 19 | South East Asian Aquarium | Singapore | Singapore |
| 20 | Sun Yat-Sen University | Guangzhou | China |
| 21 | The George Washington University | Washington D.C | USA |
| 22 | Third Institute of Oceanography, State Oceanic Administration | Xiamen | China |
| 23 | TropWATER, James Cook University | Queensland | Australia |
| 24 | United States Department of Agriculture | Washington D.C | USA |
| 25 | Yellow Sea Fisheries Research Institute, Chinese Academy of Fishery Sciences | Qingdao | China |
