# Supplementary material for: Fish-T1K (Transcriptomes of 1,000 Fishes) Project: large-scale transcriptome data for fish evolution studies
Source: Gigascience. 2016 May 3;5:18. doi: 10.1186/s13742-016-0124-7 (PMC4853854; doi:10.1186/s13742-016-0124-7)
Supplement: Additional file 4: — Transcriptome data of five species for quality control. (DOCX 20 kb) [file 13742_2016_124_MOESM4_ESM.docx]

**Additional file 4.** Transcriptome data of five species for quality control.

| **Scientific Name** | **Common Name** | **Tissue** | **BioSample ID** | **Accession No.** |
| --- | --- | --- | --- | --- |
| *Erpetoichthys calabaricus* | Reedfish | a mixture of gill/brain/liver/ovary | SAMN04577689 | SRX1661499 |
| *Erpetoichthys calabaricus* | Reedfish | gill | SAMN04577690 | SRX1661497 |
| *Erpetoichthys calabaricus* | Reedfish | liver | SAMN04577691 | SRX1661498 |
| *Megalops cyprinoides* | Indo-pacific tarpon | muscle | SAMN04577692 | SRX1665346 |
| *Megalops cyprinoides* | Indo-pacific tarpon | liver | SAMN04577693 | SRX1665345 |
| *Megalops cyprinoides* | Indo-pacific tarpon | gill | SAMN04577694 | SRX1665344 |
| *Megalops cyprinoides* | Indo-pacific tarpon | brain | SAMN04577695 | SRX1665343 |
| *Anguilla japonica* | Japanese eel | brain | SAMN04577696 | SRX1667908 |
| *Anguilla japonica* | Japanese eel | liver | SAMN04577697 | SRX1667910 |
| *Anguilla japonica* | Japanese eel | muscle | SAMN04577698 | SRX1667911 |
| *Anguilla japonica* | Japanese eel | gill | SAMN04577699 | SRX1667909 |
| *Anguilla japonica* | Japanese eel | gonad | SAMN04577700 | SRX1667912 |
| *Polyodon spathula* | American paddlefish | gonad | SAMN04577701 | SRX1665365 |
| *Polyodon spathula* | American paddlefish | muscle | SAMN04577702 | SRX1665367 |
| *Polyodon spathula* | American paddlefish | liver | SAMN04577703 | SRX1665366 |
| *Polyodon spathula* | American paddlefish | brain | SAMN04577704 | SRX1665363 |
| *Polyodon spathula* | American paddlefish | gill | SAMN04577705 | SRX1665364 |
| *Scleropages formosus* | Asian arowana | a mixture of muscle/brain/ovary/liver/gill | SAMN04577706 | SRX1668427 |
| *Scleropages formosus* | Asian arowana | ovary | SAMN04577707 | SRX1668428 |
| *Scleropages formosus* | Asian arowana | skin | SAMN04577708 | SRX1668429 |
| *Scleropages formosus* | Asian arowana | brain | SAMN04577709 | SRX1668426 |
| *Scleropages formosus* | Asian arowana | liver | SAMN04577710 | SRX1668430 |
| *Scleropages formosus* | Asian arowana | gill | SAMN04577711 | SRX1668431 |
| *Scleropages formosus* | Asian arowana | muscle | SAMN04577712 | SRX1668432 |
